# Analysis of Milk Microstructure Using Raman Hyperspectral Imaging

**DOI:** 10.3390/molecules28062770

**Published:** 2023-03-19

**Authors:** Anastasiia Surkova, Andrey Bogomolov

**Affiliations:** Department of Analytical and Physical Chemistry, Samara State Technical University, Molodogvardeyskaya Street 244, 443100 Samara, Russia

**Keywords:** milk quality, Raman spectroscopy, confocal Raman microscopy, hyperspectral imaging, principal component analysis, multivariate curve resolution, spectral clustering

## Abstract

Optical spectroscopic analysis of the chemical composition of milk in its natural state is complicated by a complex colloidal structure, represented by differently sized fat and protein particles. The classical techniques of molecular spectroscopy in the visible, near-, and mid-infrared ranges carry only bulk chemical information about a sample, which usually undergoes a destructive preparation stage. The combination of Raman spectroscopy with confocal microscopy provides a unique opportunity to obtain a vibrational spectrum at any single point of the sample volume. In this study, scanning confocal Raman microscopy was applied for the first time to investigate the chemical microstructure of milk using samples of various compositions. The obtained hyperspectral images of selected planes in milk samples are represented by three-dimensional data arrays. Chemometric data analysis, in particular the method of multivariate curve resolution, has been used to extract the chemical information from complex partially overlaid spectral responses. The results obtained show the spatial distribution of the main chemical components, i.e., fat, protein, and lactose, in the milk samples under study using intuitive graphical maps. The proposed experimental and data analysis method can be used in an advanced chemical analysis of natural milk and products on its basis.

## 1. Introduction

Rapid and reliable analysis, monitoring, and control of milk quality are important for any dairy or milk processing plant. One of them is the capability to carry out quality monitoring in real time. Numerous papers have been reported on the advantages, peculiarities, and pitfalls of using spectroscopic methods for milk quality assessment [1,2,3,4,5,6].

It is well known that milk is an emulsion of fat particles (globules) in milk plasma [7]. The plasma itself has a colloidal structure with different proteins (primarily whey protein and casein) and minerals in water. The protein molecules tend to form small particles called micelles with a characteristic size of 40–300 nm. Fat globules typically range in size from 1000 to 15,000 nm; their variability depends on many biological and technological factors. Homogenization significantly reduces the average size of fat globules and also reduces their variability [8]. The surface layer of fat particles mainly consists of phospholipids, proteins, and water, and its exact composition directly influences the stability of globules [7]. 

Colloidal particles of milk are responsible for strong light scattering, especially observed in visible and near-infrared (NIR) spectra. This effect used to be considered a hindrance to spectral analysis. However, as shown in recent works by authors, spectral information about the quantity of scattering milk particles can be used to determine milk fat and protein content [9,10,11]. Simplified analyzers based on image analysis [12,13] of the light scattering effect at different wavelengths have been proposed. However, further development of the spectroscopic analysis of dairy products requires careful studies of milk microstructure, i.e., the size distribution of colloidal particles, their shapes, and chemical composition. This information is also very important for a deeper understanding of the biochemistry of milk in general [8].

Raman spectroscopy is increasingly used for qualitative and quantitative analysis in food quality control [14,15,16,17,18,19,20]. It can be useful to analyze different types of milk (raw, pasteurized, homogenized, and skimmed) [17], as well as whole-milk powder [18]. This method was shown to be well suited for the quantification of fat [17] and total protein [14] in milk products. An advantageous combination with confocal microscopy allows laser excitation and subsequent detection of the weak Raman effect from very small sample areas, that is, with a diameter down to less than a micron. Individual spectra of bovine milk fat globules of different sizes from 1000 to 20,000 nm were obtained and studied using confocal Raman microscopy [21]. The technique of confocal microscopy in combination with Raman spectroscopy [21] or without it [22] has been successfully used to study fat globules and their membranes [22], as well as for the detection of milk adulteration [20]. The differences between human, bovine, and caprine milk fat globules can also be determined using confocal Raman microscopy [23]. No reports on using the scanning microscopy option, available in some modern devices [24], in milk analysis have been found in the literature. 

In this work, hyperspectral images (HSIs) of various milk samples were obtained using scanning confocal Raman microscopy and examined using multivariate data analysis (also known as chemometrics). Unlike the traditional approach, in which individual spectra of sample parts are taken and studied, HSIs are obtained for a selected scene and analyzed as a whole. This paper describes the methodological aspects of acquiring and analyzing the microscopic HSIs of milk and reports on practical results obtained for several representative samples of different compositions.

## 2. Results and Discussion

### 2.1. Interpretation of Spectral Signals

Raman spectroscopy measures a very weak effect of inelastic scattering that accompanies laser-induced excitation of molecules. The Raman effect is related to changes in the polarizability of molecules due to the vibration energies of functional groups [25], which results in a shift of the photon energies from the frequency of the excitation source. The major advantage of Raman spectroscopy is its ability to obtain a chemical fingerprint of a sample that contains fundamental frequencies from the mid-infrared range by means of visible and NIR light, which is transmitted by glass materials, and it has a higher energy and deeper penetration [25].

In confocal Raman microscopy, the excitation light is focused by a microscope optical system into a spot of down to 1 µm size, allowing obtaining the spectra of very small areas, thus reflecting the local chemical composition of microstructural elements of inhomogeneous samples [26]. The microscope in this case is supplemented with a high-precision motorized sample stage. It allows two-dimensional scanning of the sample to obtain the individual spectra in the chosen exact locations or an entire HSI for a pre-defined plane within a sample. 

**Figure 1:**
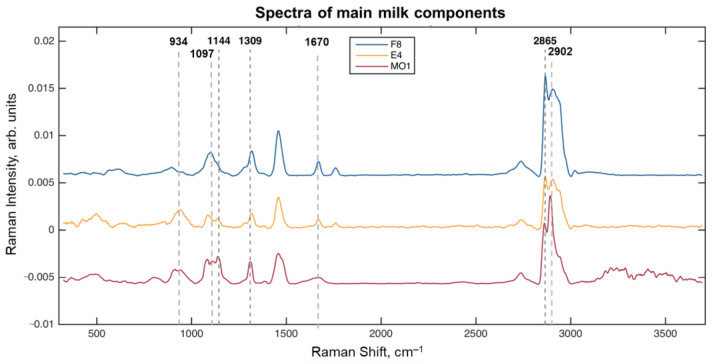
Spectra of the main milk components.

The preprocessed spectra of the milk components (Table 1) are shown in Figure 1. It should be noted that although the spectra, hereinafter called ‘fat’ (F8), ‘protein’ (E4), and ‘lactose’ (MO1), contain a mixture of signals, they correspond to the maximum of the relative concentration of the corresponding component in the sample. A preliminary interpretation of the spectra has shown several important signals. The peak at 1144 cm^–1^ (polysaccharides C–O bond stretching [27]) should be attributed to lactose, and it has the highest relative intensity in the MO1 sample medium. Two other characteristic peaks of lactose occur at 934 cm^−1^ and 1097 cm^−1^, associated with the C–C and C–O vibrations [27]. A standalone peak at 1309 cm^–1^ is present in all spectra in Figure 1, because it belongs to the twisting vibrations of the common CH_2_ group [17,18,20,21]. A few overlapping peaks are observed in the range from 2800 cm^–1^ to 3000 cm^–1^. The intensive peak at 2865 cm^–1^ can be assigned to the symmetric C–H stretching vibrations of CH_2_ [17,18,20,21], mainly of fatty acids. This conclusion is confirmed by the highest intensity of this signal in the F8 spectrum, taken within the fat globule. The peak at 2902 cm^–1^ is the strongest in the spectrum of MO1, and therefore, it can be caused by the C–H stretching of the eight methine groups of lactose [27]. No clear and selective peak of proteins was found. Although the weak peak around 1670 cm^−1^ could belong to the amide I bands of protein [18], it can hardly be seen below the stronger signal of fatty acids that occurs in the same region [28].

**Figure 2:**
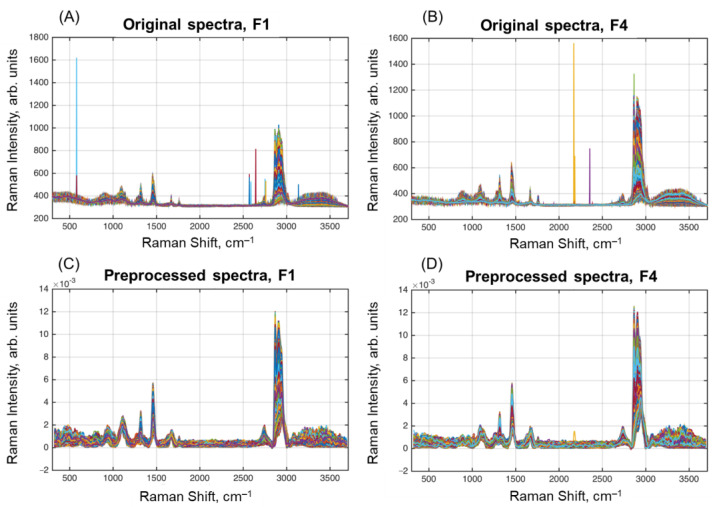
Original (**A**,**B**) and preprocessed (**C**,**D**) spectra of the samples F1 (**A**,**C**) and F4 (**B**,**D**).

Several milk samples were selected for further HSI analysis. In the present paper, we report on results obtained for Samples F1 and F4 (Table 1). The raw and preprocessed spectra for the samples are shown in Figure 2. As expected, the spectra show mostly the same peaks as in the selected spectra of the milk components. Moreover, a broad water peak is clearly seen at 3100–3500 cm^–1^. 

### 2.2. Exploratory Analysis of Hyperspectral Images

**Figure 3:**
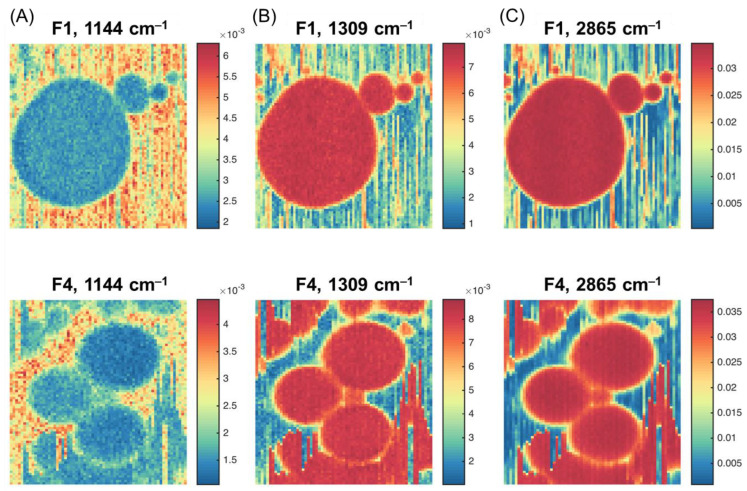
Hyperspectral images for Samples F1 and F4 at the selected wavelengths: (**A**) 1144 cm^−1^; (**B**) 1309 cm^−1^; (**C**) 2856 cm^−1^.

Pseudo-color images (Figure 3) were built for the chosen samples (F1 and F4) using the Raman spectral intensities for the variables selected in Figure 1. The color gradient reflects the relative intensity of a particular peak, from the weakest (dark blue) to the strongest (dark red). It can be clearly seen that the highest contrast was obtained for the peaks corresponding to lactose and fat.

The images in Figure 3B show the Raman intensity at 1309 cm^–1^ related to the vibrations of the CH_2_ group that is abundant in fatty acids [17,18,20,21]. The ability of confocal microscopy to take the spectra of a small volume within the fat globule makes it possible to obtain an almost pure spectrum of milk fat, although the lactose and protein concentration in the bulk sample is high (Table 1). Therefore, Figure 3B describes the overall distribution of milk fat. Indeed, the maximum intensity is observed for fat globules, which are clearly distinguished in the image as a set of round objects. The vertical ‘scratches’, perhaps, result from the effect known as ‘optical tweezers’—the ability of a highly focused laser beam to capture and move small particles (e.g., smaller fat globules and protein micelles) during the scanning. This effect is widely used to hold and fix small objects for spectral measurements, e.g., in cell biology [29]. In our case, however, it is a parasitic phenomenon that reduces the quality of spectral images. It is clearly visible, especially for Sample F4, which contained smaller fat globules.

The images in Figure 3A (at 1144 cm^–1^) show the highest intensities in the liquid phase around the fat globules, which confirms the assignment of this peak to lactose.

The images in Figure 3C for the peak maximum at 2865 cm^–1^ appear very similar to the images taken for 1309 cm^–1^. However, the difference in intensities between fat globule pixels and their surroundings is smaller, so the presence of both fat and lactose can be expected, because of the band overlap, as discussed in Section 2.1.

### 2.3. Principal Component Analysis and Spectral Unmixing

Principal component analysis (PCA) is a powerful method for exploring multivariate datasets that have significant collinearity, such as spectral data [30]. Visual investigation of intensity maps for individual spectral variables is a very powerful research tool, if the spectra have clear, non-overlapping peaks for each of the chemical components. Signal overlap resulting from insufficient spectral or spatial resolution, which is a common situation in the spectral analysis of complex mixtures, strongly complicates the data analysis and can even lead to wrong conclusions. Effective use of spectral information for better interpretation results can be reached by the application of factor spaces and projection techniques provided by the arsenal of chemometrics. PCA is a common and natural tool for the exploratory analysis of multivariate data. PCA performs a decomposition of the spectral data, such as HSIs, into a linear combination of principal components. The PCs can then be investigated and interpreted one by one. 

**Figure 4:**
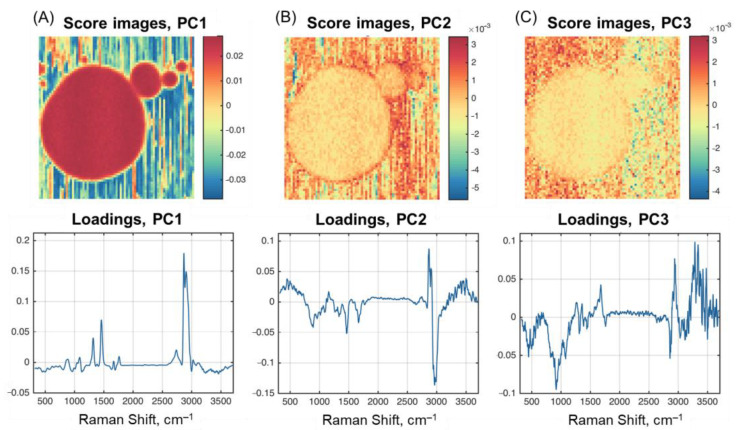
PCA scores and loadings for Sample F1: (**A**) PC1; (**B**) PC2; (**C**) PC3.

As described in Section 3.4.1, the investigation of PCA results obtained for HSI can be performed using images of the scores carrying spatial information and line plots of the loadings (spectral information) for individual PCs. Figure 4 shows the scores and loadings for the first three PCs calculated from the HSI of Sample F1. The results obtained for Sample F4 are very similar and will not be shown. The loadings in this case are the variable weights: each wavelength contributes to a particular PC. The scores are data projections for individual PCs. The higher the absolute intensity of the variable in loadings (values along the wavenumber axis), the higher its contribution to the pixel intensities in the score image. Therefore, if a loading plot has a spectrum-like pattern that has specific spectral features of a particular component, the scores can be similarly interpreted as a concentration map of the respective component. 

As one can notice, the loading plot for the first PC (Figure 4A) is very similar to the spectra obtained for fat (Figure 1), with slight deviations in the form of negative peaks in the water absorption region. The score image supports this assumption very well by revealing a contrast image of the fat globules (including the optical tweezers effect for smaller particles). The image closely resembles that obtained for the peak at 2865 cm^–1^ (Figure 3).

The results for the second and third PCs are more difficult to interpret. PC2 and PC3 in Figure 4 explain the residual variance in the data, which is left after subtracting the variance captured by previous components. PC2 loadings contain negative peaks, the first of which is typical for fat and observed at 1670 cm^–1^. The peak at 1460 cm^–1^ exists for all three milk components. 

The loading plot for PC2 shows various spectral features indicating its relation to different milk components. The area of C-H stretching vibrations between 2800 cm^–1^ and 3000 cm^–1^ differs partially. Two positive peaks at 2865 cm^–1^ and 2902 cm^–1^ and a negative signal around 2960 cm^–1^ can point to structural differences of aliphatic groups included in fat and protein molecules. All peaks characteristic for fat (Section 3.4.1) are reasonably present in PC1 loadings. The respective score image for PC2 exhibits higher intensity values on the edge of fat globules including the area, where the globules contact with each other. These facts can be explained by the presence of a stabilizing protein-based membrane around the globule [22]. The third PC looks very noisy and experiences the largest contribution from the broad water absorbance at the high-wavenumber part of the spectra.

PCA is quite efficient in revealing all kinds of systematic variation in spectral data, e.g., groups, trends, and outliers. However, PCs are abstract factors that do not necessarily have any physical or chemical interpretation. 

### 2.4. Multivariate Curve Resolution

Spectral unmixing [31,32] can tackle the above drawbacks of PCA by performing similar linear data decomposition, but results in the latent variables having direct chemical interpretation, i.e., pure-component spectra and complementary concentration maps. This can be achieved by using prior knowledge of the system, expressed in terms of constraints used by the resolution algorithm. 

**Figure 5:**
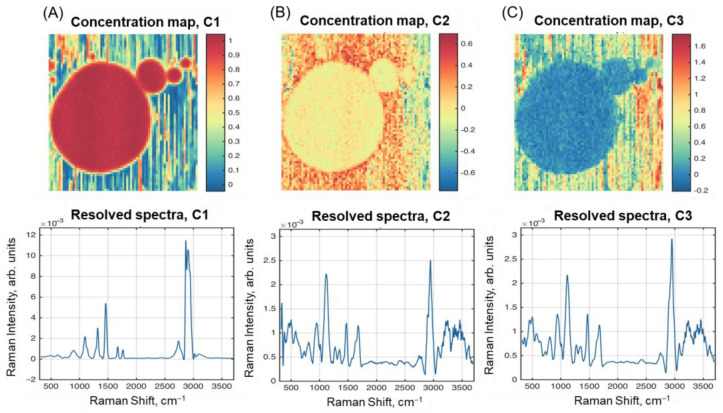
Results of spectral unmixing with SIMPLISMA: resolved concentration maps and spectra of: (**A**) Component 1; (**B**) Component 2; (**C**) Component 3.

Figure 5 presents resolved spectra and concentration maps obtained using SIMPLISMA curve resolution for Sample F1. The first resolved spectrum and HSI (C1 in Figure 5A) closely resemble the fat spectrum (F8 in Figure 1) and first PC loadings (Figure 4A). The concentration map for C1 is also very similar to both the PC1 scores and the raw intensity map for the peak at 2865 cm^–1^ (Figure 3C). The resolved spectra for the other two components in Figure 5B,C look similar to each other, as well as to the spectrum of Sample E4 in Figure 1 (combination of protein and lactose). 

It should also be noted that the concentration maps for C2 and C3 look complementary, so that C3 explains the residual variation after removing the contribution of C1 and C2. Therefore, we can conclude that the SIMPLISMA algorithm, with the standard settings used here, failed to resolve the three chemical components of milk in the spectral data.

**Figure 6:**
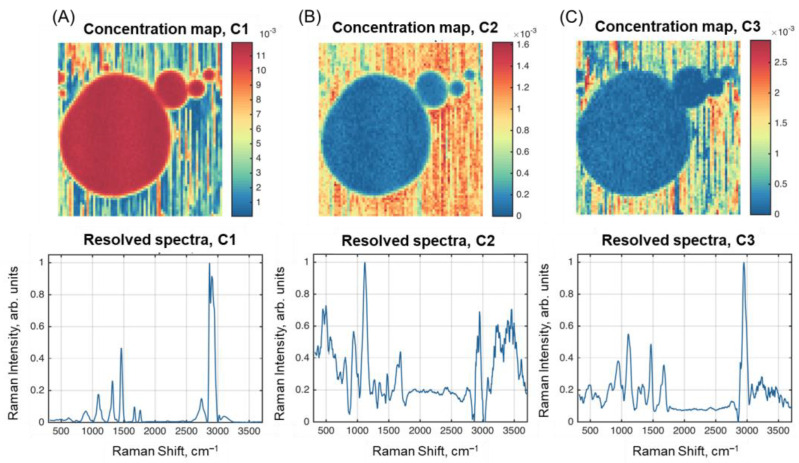
Results of spectral unmixing with ALS-MCR: resolved concentration maps and spectra of: (**A**) Component 1; (**B**) Component 2; (**C**) Component 3.

The results of spectral unmixing obtained with the ALS-MCR algorithm are shown in Figure 6. The resolved spectra are quite similar to the ones obtained by SIMPLISMA. However, the concentration maps for C2 and C3 show better contrast between the three parts.

**Figure 7:**
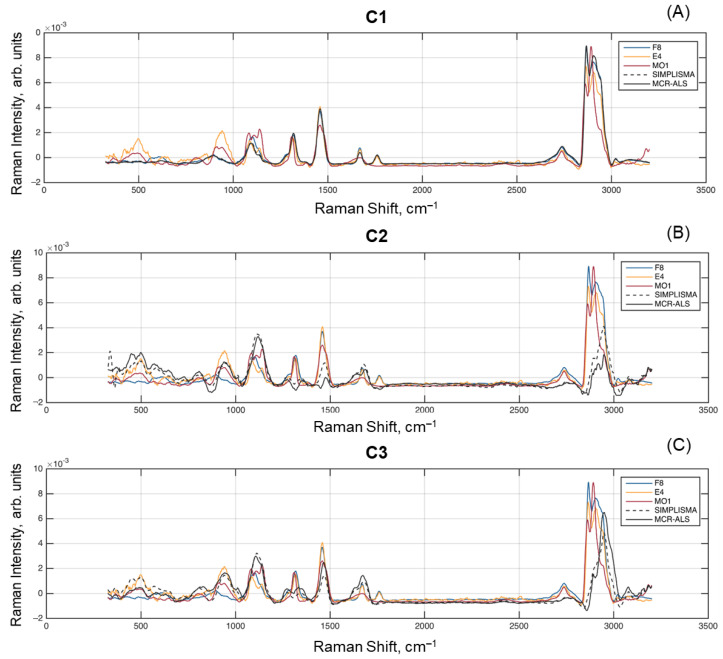
Comparison of spectra resolved by multivariate curve resolution methods and measured reference spectra: (**A**) Component 1; (**B**) Component 2; (**C**) Component 3.

Figure 7 shows the resolved spectra for each of the spectral unmixing methods, as well as the reference spectra F8, E4, and MO1 from Figure 1. All spectra were subject to subtracting the mean value, removing baseline offset, and performing the unit area normalization. It is clear that both resolved spectra for C1 are almost identical to each other as well as to the spectrum of sample F8 (fat). At the same time, the C2 and C3 spectra show some differences, e.g., the spectrum obtained for C2 by SIMPLISMA has larger peaks at 2940–2950 cm^–1^, which is typical for both fat and protein spectra present in the globule surface.

### 2.5. Spectral Clustering

**Figure 8:**
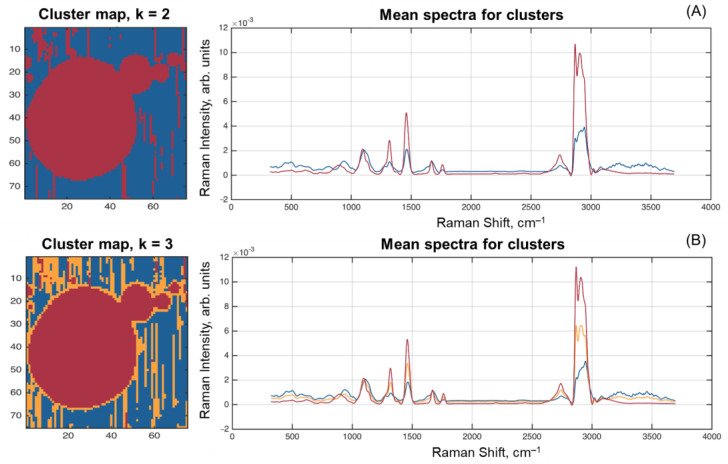
Spectrum clustering results for two (**A**) and three (**B**) clusters.

Finally, the spectra from F1 and F4 HSI were clustered by the K-means method [33] using two and three clusters. The corresponding cluster maps and mean spectra for each cluster are shown in Figure 8.

The results of clustering into two clusters expectedly split the pixels to those belonging to the fat globules (including the particles moved by laser) and to the area around them. The mean spectra of the clusters clearly resemble the spectra for F8 (fat) and MO1 (lactose) samples.

However, applying the algorithm that splits pixels into three clusters gave quite an interesting result—pixels on the edge of fat globules, as well as some of them moved by the laser beam, formed the third cluster. The mean spectrum of this cluster looks very similar to the spectrum of fat with smaller intensities and several minor differences, like in the range 800–1200 cm^–1^, where it looks closer to the spectrum of the second cluster. It can also be seen that it looks very close to the spectrum obtained for Sample E4, which mainly represents a combination of lactose and protein. At the same time, the image in Figure 8B allows one to suggest that the mean spectrum of this additional cluster (orange curve in Figure 8B) reflects the composition of the protein membrane shell [22] surrounding the fat globule.

Additional chemical and spectral information on the milk constituents and their spatial distribution opens the possibility to use multivariate curve resolution algorithms to deconvolve the pure-component contributions from the complex HSIs of their mixtures. On the one hand, the resolved components facilitate the interpretation of the vibrational spectra of individual chemical substances in terms of constituting functional groups. HSI gives a possibility to explore two-dimensional intensity maps made for particular spectral variables, and consequently, for particular components. Moreover, the use of chemometric methods, such as PCA, spectral unmixing, and spectral clustering, allows acquiring even more information about the analyzed samples, for example, to investigate the composition of fat globules in-depth. 

## 3. Materials and Methods

### 3.1. Milk Samples

Five frozen samples of natural milk and its derivatives (commercially available from QSE GmbH, Germany) were used in this study. Prior to the analysis, the samples were slowly melted at 43 °C for an hour, as recommended by the supplier. The selected samples had strongly varying content of fat, protein, and lactose. The main data on the sample composition and spectra acquisition are presented in Table 1. Fat (F8)-, protein (E4)-, and lactose-reach (MO1) milk samples were used to obtain possibly pure spectra of the respective components to be subsequently used for chemical interpretation and data analysis. Milk samples F1 and F4 were used for the acquisition of HSI.

### 3.2. Spectra Acquisition and Preprocessing

Spectroscopic measurements were carried out on a milk drop with a volume of about 1 μL. The drop was placed on a glass slide and protected by a quartz coverslip. The resulting spot of milk between the two glasses had a diameter of about 10 mm and a thickness of about 10 μm. The distance between the glasses was measured in a separate experiment using the scanning mode of the instrument. 

Individual Raman spectra and HSI were acquired using the Alpha300 RS Raman—Scanning Near-Field Optical Microscopy (SNOM) by WITec (Ulm, Germany). A green He-Ne laser with a band maximum at 532 nm and a power of about 100 mW was used as the excitation source. Raman spectra were collected using a 600 g/mm diffraction grating and an electron multiplying charge-coupled device (EMCCD) camera (DU970_BV35) with a chip size of 1600 × 200 pixels. Raman spectra ranging from 0 to 3709.8 cm^−1^ were recorded with an integration time of 0.1 s without accumulation. For the representation and data analysis, the spectra were limited to the region of 400–3600 cm^−1^. Hyperspectral images were taken with a spatial resolution of about 0.8 µm and a spectral resolution of 1 cm^−1^. Individual spectra were obtained by focusing the laser beam at various points of an enlarged area of the sample. Hyperspectral images were acquired by a point-by-point scanning of a selected plane within the sample. The scanning depth and sample area for hyperspectral analysis (about 60 × 60 μm) were preliminary chosen by visual investigation under a 50×-magnification objective. Exact positioning and the main analysis were performed through a 63×/1.2 W objective. Although only four milk types (Table 1) were used in this study, each of them was analyzed many times by taking a new drop for the microspectroscopic measurement. A variable size of the scanning area for HSI was used depending on the sample. For the HSI discussed here, the image sizes were: 15 × 15 μm for F1 and 16 × 13 μm for F4. The most representative spectra and HSIs were selected for the discussion.

Data were acquired using instrumental software (WITec Project, version 2.08). Prior to the data analysis, the spectra were preprocessed. Cosmic spikes (narrow intensive peaks in Raman spectra caused by high-energy photons hitting the highly sensitive CCD detector) were eliminated using an advanced median filter [34,35]. Baseline was corrected using an automated asymmetric least-squares (AsLS) algorithm [36]. The spectral noise was reduced by applying a Savitzky–Golay filter [37]. Spectral intensity variations (caused by the random nature of the Raman signal in a non-homogeneous medium) were eliminated at the final step of the preprocessing by means of normalization to the unit area by dividing each variable by the sum of absolute values of all selected variables for the given spectrum [38,39]. Data preprocessing algorithms were implemented in a homemade script for MATLAB by MathWorks Inc. (Natick, MA, USA). The preprocessing parameters (if any) were optimized manually by visual investigation of spectra and hyperspectral data. 

### 3.3. Representation of Hyperspectral Images

An HSI is a hypercube with two spatial (X, Y) dimensions and one spectral (Z) dimension. Thus, if *H* points were measured along Y, *W* points, along X, and every spectrum has *M* values (wavenumbers), then the data form a *W* × *H* × *M* array. Each pixel of an HSI contains an individual spectrum of the depicted area. HSIs can be processed and analyzed using the conventional spectroscopy methods, which consider the data as a bunch of *N* = *W*·*H* spectra without considering the spatial information. Alternatively, HSIs can be analyzed using the methods of image analysis, considering the dataset as *M* images of *W* × *H* pixels each.

In this study, obtained HSIs were represented as two-dimensional intensity maps with a pseudo-color scale of the pixel intensity showing the value assigned (e.g., relative spectral intensity at a chosen wavelength or a multivariate parameter).

### 3.4. Data Analysis

#### 3.4.1. Principal Component Analysis

PCA [30] was used for the initial exploratory analysis of the internal data structure of hyperspectral Raman images. PCA helps to estimate the data complexity in terms of the main *A* factors called principal components (PCs). PCA starts from the decomposition of raw data matrix **X** (*N* × *M*) into two complementary matrices of scores **T** (*N* × *A*) and loadings **P** (*M* × *A*):

**X** = **TP**^T^ + **E** (1)

The product **TP**^T^ is a projection of the data into a new space of principal components. The matrix **E** (*N* × *M*) of residuals contains irrelevant information (the noise) that stays outside the PC-space.

The matrix of scores **T** contains the main information about the samples, i.e., HSI pixels in our case. Therefore, the values of orthogonal score vectors t*_i_* (*i* from 1 to A) composing **T** can be represented as pseudo-color images suitable for interpreting the portion of variance explained by the respective PC. On the other hand, the loadings **P** contain the coordinates of the unit vectors of PC-axes in the initial X-variable space. The loading vectors **p**_i_ being represented as line plots along the spectral axis of wavenumbers carry interpretable information on the spectral features behind the PC-factors. Therefore, the score plots show a relationship among the HSI pixels, projected to the PC-space, whereas the loadings reveal the influence (or weight) of original variables. Joint use of the score and loading plots is an efficient tool for exploring patterns and trends in complex multivariate data, as well as the relationship between the objects and variables. 

To apply PCA to HSIs the hypercube should be unfolded into a matrix (*W*·*H* × *M*), where the rows are pixels and the columns are wavenumbers. After PCA decomposition, the matrix of scores can be refolded by giving the spatial relationship back to the values of scores.

#### 3.4.2. Multivariate Curve Resolution (Spectral Unmixing)

Spectral unmixing or multivariate curve resolution (MCR) is stated for a group of methods aiming at the decomposition of original spectral data matrix **X** (*N* × *M*) into the spectra of pure chemical components and a corresponding concentration map [40]. Assuming that the original spectra can be described by the bilinear additive model, the decomposition can be written in matrix form (Equation (2)):

**X** = **CS**^T^ + **E** (2)

where **S** (*M* × *A*) is a matrix of resolved pure-component spectra, **C** (*N* × *A*) is a matrix of concentration values, and **E** (*N* × *M*) is an error matrix. For the hyperspectral data, concentration matrix **C** can be refolded into a three-way array (*H* × *W* × *A*), where *A* is the number of pure components, and represented as concentration maps, i.e., pseudo-color images, where the color gradient is used to show relative concentrations of a selected pure component for each pixel (similar to the score images in PCA, Section 3.4.1).

The bilinear additive model expression (Equation (2)) resembles PCA decomposition (Equation (1)). However, in the former case, there is no orthogonal requirement to the matrix **S** and the principle of maximum variation does not apply. Finding **C** and **S** from the mixture spectra **X** is an inverse problem, and in the general case, it gives a range of solutions. 

Two methods of mathematical spectral unmixing were used in this study. One of them, called simple-to-use interactive self-modeling mixture analysis (SIMPLISMA) by Windig et al. [31,32,41], is based on the so-called pure (or purer) spectral variables, i.e., the wavenumbers mainly influenced by only one of the mixture components. Finding the purest variables one by one, one can reconstruct the pure-component spectra using the least-squares regression algorithm.

Another approach to solving the problem of spectral unmixing is the alternating least-squares–multivariate curve resolution (ALS-MCR) algorithm. It makes use of initial estimates of the pure spectra (for instance, known spectra of the components or PCA loadings). The solution is found iteratively until the best fit to spectral data is attained [42]. To reduce the ambiguity of the ALS-MCR solution, non-negativity constraints were applied to the spectra and concentrations [43]. 

#### 3.4.3. Clustering of Spectra

Clustering is a multivariate classification algorithm. In the present study, it was used to arrange HSI spectra into several groups (clusters) according to their similarity. Euclidean distance between spectra in a multidimensional spectral space was used as a similarity criterion. The idea of using clustering was to find spectra of similar objects (for instance, pixels within fat globules) without a priori knowledge, and combine them together for further analysis.

One of the simplest clustering algorithms called K-means [33] was used in this study. This algorithm aims at finding K clusters in the spectral space so that the sum of squared distances between the mean spectrum and all individual spectra within a particular cluster is minimal. The algorithm is iterative and works as follows:

(1) Assign K-mean points (centroids of clusters) in the spectral space randomly.
(2) Calculate distances from each spectrum to each mean point.
(3) Assign each spectrum to the proper cluster by selecting a minimal distance.
(4) Calculate a new mean for each cluster by averaging the assigned spectra.

Steps 2–4 are repeated until the convergence is reached, i.e., when the cluster mean positions stop changing.

The K-means clustering algorithm, applied to HSI spectra in this study, results in new cluster maps, where the pixels are colored depending on a particular cluster they belong to. The unfolding/refolding procedure (as in Section 3.4.1 and Section 3.4.2) is applied to analyze three-dimensional data using the K-means algorithm. Moreover, the mean spectra of the corresponding clusters can be interpreted in terms of the chemical composition of the respective image elements.

## 4. Conclusions

This feasibility study has shown that scanning confocal Raman microscopy is a powerful tool for the investigation of the chemical composition of natural milk without the need to destroy the colloidal structure of samples. The main components of milk responsible for its nutritional value can be clearly identified in the Raman spectra. The problem of signal overlap in the spectra can be overcome by mathematical spectral unmixing based on chemometric methods of curve resolution. 

This study has proved that the correct application of chemometrics can significantly increase the value of hyperspectral Raman imaging, particularly for dairy analysis. The ALS-MCR algorithm with non-negativity constraints has shown to be well suited for the analysis of individual components. HSIs can be deconvoluted into colored spectral maps indicating areas reach of fats, proteins, and lactose. Such representation facilitates qualitative microstructural analysis of various samples. 

Research efforts should be aimed at further improvement of the method. Thus, the higher magnification of the microscope and the respective higher image resolution are expected to enable the analysis of individual protein micelles and other particles, such as somatic cells. The use of three-dimensional hyperspectral Raman images should help to obtain a detailed description of the fat globule structure at the molecular level, particularly to investigate the structure of its stabilizing surface membrane. Further improvement of the sample preparation technique and data analysis algorithms will make it possible to identify minor components of milk. Qualitative and quantitative characterization of the liquid media is necessary to obtain a complete chemical description of the milk microstructure. 

## Figures and Tables

**Table 1:** Description of milk samples and spectral data acquired.

Sample	Fat, %	Protein, %	Lactose, %	Data Acquired
F1 milk	2.3	3.4	4.9	HSI of globule area
F4 high-fat milk	5.57	3.62	4.72	HSI of medium area
E4 high-protein milk	4.5	4.1	5.3	medium spectra: protein
MO1 skim milk	0.1	0.5	3.6	medium spectra: lactose
F8 cream	16.1	3.0	23.9	globule spectra: fat

## Data Availability

Not applicable.

## Sample Availability

Data are available from the authors on request.
